# Treatment resistant trigeminal neuralgia relieved with oral sumatriptan: a case report

**DOI:** 10.1186/1752-1947-3-7229

**Published:** 2009-05-08

**Authors:** JA Moran, A Neligan

**Affiliations:** 1 Department of General Practice, Brookfield Health Sciences Centre, University College Cork, Cork, Ireland; 2 UCL Institute of Neurology, 33 Queens Square, London, WC1N 3BG, UK

## Abstract

**Introduction:**

Treatment-resistant trigeminal neuralgia is a distressing condition, for both the patient and the treating doctor. To our knowledge, there are no reported cases of trigeminal neuralgia successfully treated with oral sumatriptan in the literature.

**Case presentation:**

A 51-year-old Caucasian woman was prescribed opiate analgesia for management of her treatment-resistant trigeminal neuralgia. Given the possible harmful effects of initiating such a course of treatment, a speculative therapeutic trial with oral sumatriptan was initiated with a successful outcome.

**Conclusion:**

This case raises the hypothesis that oral sumatriptan may be an effective drug in the treatment of trigeminal neuralgia. Further research is required to test this theory.

## Introduction

One of the challenges in primary care is the ongoing management of patients with chronic painful syndromes who have not responded to the standard treatment regimens. Polypharmacy, multiple speciality referrals, patient dissatisfaction, depression, frequent consultations and physician frustration often ensues [1]. Such conditions tend to have a high morbidity and low mortality. Trigeminal neuralgia is a typical example of such a condition. We present the case of a middle-aged woman who, despite neurosurgical decompressive surgery, radiofrequency ablation therapy, as well as multiple speciality referrals including dentistry and a specialist pain clinic review, continued to experience frequent, severe and disabling episodes of right-sided facial pain, attributed to trigeminal neuralgia. Her symptoms rapidly responded to a trial of oral sumatriptan 50 mg and are currently well controlled with intermittent sumatriptan and maintenance propranolol.

This case highlights a further possible treatment option for trigeminal neuralgia resistant to standard treatment options.

## Case presentation

We present the case of a 51-year-old Caucasian woman with a four-year history of recurrent, right-sided facial pain, in the distribution of all three branches of the trigeminal nerve. These episodes occurred on average two to three times per week and were characterised by brief episodes of unilateral right-sided sharp lancinating pain, lasting on average less than one minute and predominantly affecting the maxillary and mandibular divisions of the trigeminal nerve, although occasionally involving all three branches. Attacks were typically triggered by such actions as washing her face or smiling. There were no other features associated with these attacks; in particular there were no associated headaches or visual disturbance. She had no prior history of headache and there was no family history of trigeminal neuralgia. Her neurological and dental examinations, a computed axial tomography scan of brain and sinuses, and routine blood tests were all normal.

The patient initially commenced on gabapentin (up to 1200 mg daily) to which carbamazepine (up to 800 mg daily) was later added without any significant benefit. Following a neurosurgical assessment, she underwent decompression of her right trigeminal nerve, nine months after the initial onset of her symptoms. Unfortunately, her symptoms worsened following this and she was referred for radiofrequency ablation 14 months after the decompressive surgery. This resulted in some permanent numbness in her right infra-orbital area but no pain relief. She was then referred to a specialist pain clinic and initially treated with a combination of dothiepin (75 mg daily), pregabalin (600 mg daily) and numesulide (200 mg daily). This regime resulted in a transient improvement in her symptoms. At this stage, oxycodone 10 mg daily was added; a treatment escalation that her general practitioner (GP) had reservations about. Based on previous experience using sumatriptan for abdominal pain [2], a trial of oral sumatriptan 50 mg was suggested, to be taken at the onset of facial pain. This resulted in effective pain relief, and propranolol to a dose of 160 mg daily was added. This reduced the frequency of her facial pain from two to three episodes per week to one every two weeks. Numesulide, dothiepin and pregabalin were safely withdrawn.

## Discussion

Headaches and facial pain are common, and patients with these symptoms frequently present to their GP for advice. Careful history taking and physical examination are the mainstay of accurate diagnosis in the evaluation of these patients. The majority of conditions are self-limiting and managed in general practice without the need for referral or further diagnostic tests. It can, however, be difficult to distinguish between migraine, trigeminal neuralgia, atypical facial pain and cluster headaches with clinical certainty [3]. The symptoms of these conditions may overlap and they also share some common treatments [4].

Trigeminal neuralgia is important due to its high morbidity. It usually presents after the age of 50, is unilateral and affects the sensory branches of the trigeminal nerve [5]. The cause of trigeminal neuralgia is not fully understood. It is thought to be due to irritation or compression of the trigeminal nerve root by neighbouring arteries [6]. This is the basis for neurosurgical decompression procedures. Review of the current specialist literature shows that there is little evidence to support the use of sumatriptan in the management of trigeminal neuralgia. What evidence does exist describes the use of parenteral sumatriptan only [7] and involves small numbers of patients [8].

Sumatriptan is a serotonin (5-HT) agonist, specifically developed to relieve migraine headaches. Although the cause of migraine is not fully understood, it is thought that a widening of blood vessels in the brain causes the throbbing pain of migraine headaches. Sumatriptan works by causing vasoconstriction of these vessels via the stimulation of serotonin (or 5-HT) receptors. Naturally occurring serotonin normally acts on these receptors, causing blood vessels in the brain to narrow. Sumatriptan mimics this action of serotonin by directly stimulating the serotonin receptors in the brain. This results in narrowing of the blood vessels and in effective relief of the migraine headache pain. A Cochrane review has confirmed the effectiveness of sumatriptan in the acute management of migraine [9].

Propranolol, a beta-blocker, is recognised as effective medication for the prophylaxis of migraine [10] and is commonly used for this indication.

The question of diagnosis in this patient is critical, given the response to treatment. It is tempting to take the view that this patient has an atypical form of migraine, based on her response to both specific anti-migraine medication (sumatriptan) and a proven anti-migraine prophylaxis medication (propranolol), and that the original diagnosis of trigeminal neuralgia was incorrect. However, the clinical presentation of her symptoms strongly supports a diagnosis of trigeminal neuralgia, given the very brief duration of the episodes, the absence of any other symptoms other then unilateral pain in the distribution of the trigeminal nerve and the presence of clear physical triggers, such as washing her face, precipitating an attack. Moreover, a diagnosis of trigeminal neuralgia was felt to be the correct diagnosis by an experienced general practitioner, a neurologist and a neurosurgeon who advocated and carried out invasive decompressive surgery and radio-ablation which would be wholly inappropriate if any doubt had existed concerning the diagnosis. Given this, a more plausible explanation is that the vasoconstrictive mechanism of the sumatriptan relieved whatever compressive or irritative effects were occurring at the root of the affected trigeminal nerve. Such speculations highlight the fundamental problems in drawing conclusions from such 'trials of therapy'. A hypothesis has arisen and needs to be tested using more rigorous and valid research methodology.

## Conclusion

In conclusion, based on the experience of this case and a review of the current specialist literature, we advise that GPs, when treating patients with difficult to manage trigeminal neuralgia, consider a trial of oral sumatriptan and report their findings.

## Competing interests

The authors declare that they have no competing interests.

## Consent

Written informed consent was obtained from the patient for publication of this case report. A copy of the written consent is available for review by the Editor-in-Chief of this journal.

## Authors' contributions

JM was the patient's primary care physician. Both JM and AN contributed equally to the design, literature review and drafting of this case report. JM and AN have both seen and approved the final submitted manuscript.

## References

[B1] Court C New day centre hope for "heartsink" patients BMJ 1994 309 500

[B2] Moran JA Adult abdominal migraine and migraine: a case report Ir Med J 1998 91 215 216 10069134

[B3] Feinmann C Peatfield R Orofacial neuralgia. Diagnosis and treatment guidelines Drugs 1993 46 263 268 10.2165/00003495-199346020-00004 7691515

[B4] Rosen TD Antiepileptic drugs in the management of cluster headaches and trigeminal neuralgia Headache 2001 41 S25 S32 10.1046/j.1526-4610.2001.01154-5.x 11903537

[B5] Bennetto L Patel NK Fuller G Trigeminal neuralgia and its management BMJ 2007 334 201 205 10.1136/bmj.39085.614792.BE 17255614 PMC1782012

[B6] Kanai A Saito M Hoka S Subcutaneous sumatriphan for refractory trigeminal neuralgia Headache 2006 46 577 582 10.1111/j.1526-4610.2006.00405.x 16643550

[B7] Kanai A Suzuki A Osawa S Hoka S Sumatriphan alleviates pain in patients with trigeminal neuralgia Clin J Pain 2006 22 677 680 10.1097/01.ajp.0000210917.18536.0d 16988562

[B8] Al Balawi S Tariq M Feinmann C A double-blind, placebo-controlled, crossover, study to evaluate the efficacy of subcutaneous sumatriphan in the treatment of atypical facial pain Int J Neurosci 1996 86 310 319 8884400 10.3109/00207459608986720

[B9] McGrory DC Gray RN Oral sumatriphan for acute migraine Cochrane Database of Systemic Reviews 2003 CD002915 12917936 10.1002/14651858.CD002915

[B10] Linda K Rossnagel K Proranolol for migraine prophylaxis Cochrane Database of Systemic Reviews 2004 CD003225 10.1002/14651858.CD003225.pub2 15106196

